# Supplementary material for: A Global Screen for Assembly State Changes of the Mitotic Proteome by SEC-SWATH-MS
Source: Cell Syst. 2020 Feb 26;10(2):133–155.e6. doi: 10.1016/j.cels.2020.01.001 (PMC7042714; doi:10.1016/j.cels.2020.01.001)

Q8NI08 | NCOA7_HUMAN | NCOA7 ERAP140 ESNA1 Nbla00052 Nbla10993
Monomer MW [kDa]: 106.162 Monomer expected elution fraction: 40

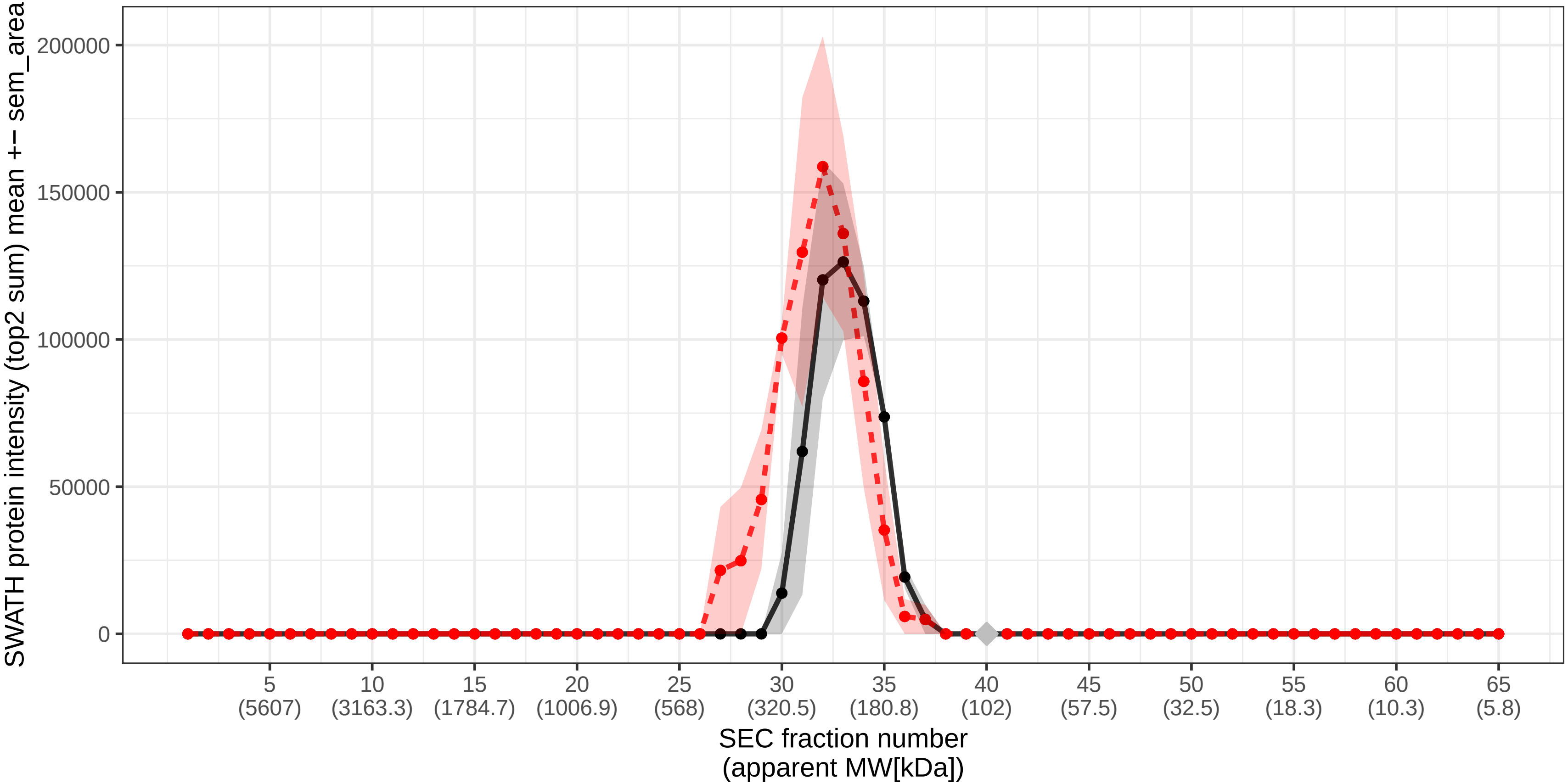

Supplement: Data S1. SEC-SWATH-MS Protein Chromatograms, Related to Figure 1 [file mmc6.zip › SECchrom_Q8NI08_NCOA7_HUMAN_NCOA7_ERAP140_ESNA1_Nbla00052_N.pdf]
